# The effect of acute malnutrition on enteric pathogens, moderate-to-severe diarrhoea, and associated mortality in the Global Enteric Multicenter Study cohort: a post-hoc analysis

**DOI:** 10.1016/S2214-109X(19)30498-X

**Published:** 2020-01-22

**Authors:** Kirkby D Tickell, Rumana Sharmin, Emily L Deichsel, Laura M Lamberti, Judd L Walson, A S G Faruque, Patricia B Pavlinac, Karen L Kotloff, Mohammod J Chisti

**Affiliations:** a Department of Global Health, University of Washington, Seattle, WA, USA; b Department of Epidemiology, University of Washington, Seattle, WA, USA; c Department of Medicine, University of Washington, Seattle, WA, USA; d Department of Pediatrics, University of Washington, Seattle, WA, USA; e Childhood Acute Illness and Nutrition (CHAIN) Network, Nairobi, Kenya; f International Centre for Diarrheal Disease Research (icddr, b), Dhaka, Bangladesh; g Center for Vaccine Development and Global Health, School of Medicine, University of Maryland, Baltimore, MD, USA; h The Bill & Melinda Gates Foundation, Seattle, WA, USA

## Abstract

**Background:**

Host vulnerabilities associated with acute malnutrition could facilitate the ability of specific enteric pathogens to cause diarrhoea and associated mortality. Using data from the Global Enteric Multicenter Study, we assessed whether acute malnutrition modifies the association between common enteric pathogens and moderate-to-severe diarrhoea, and whether associations between enteric pathogens and death were modified by acute malnutrition.

**Methods:**

Children with moderate-to-severe diarrhoea and age-matched and community-matched controls were included in this post-hoc analysis if their mid-upper arm circumference had been measured and if they were older than 6 months of age. Acute malnutrition was defined as mid-upper arm circumference below 12·5 cm, capturing both severe acute malnutrition (<11·5 cm) and moderate acute malnutrition (≥11·5 cm and <12·5 cm). We tested whether acute malnutrition modified associations between enteric pathogens and moderate-to-severe diarrhoea in conditional logistic regression models. Among children with moderate-to-severe diarrhoea, Cox proportional hazards regression evaluated the modifying effect of acute malnutrition on the relationship between pathogens and 60-day fatality rate.

**Findings:**

The age, site, and co-infection adjusted odds ratios (aORs) for moderate-to-severe diarrhoea associated with typical enteropathogenic *Escherichia coli* among children aged 6–11 months was 2·08 (95% CI 1·14–3·79) in children with acute malnutrition, and 0·97 (0·77–1·23) in children with better nutritional status, compared with healthy controls. Enterotoxigenic *E coli* producing heat-stable toxin among children aged 12–23 months also had a stronger association with moderate-to-severe diarrhoea in children with acute malnutrition (aOR 7·60 [2·63–21·95]) than among similarly aged children with better nutritional status (aOR 2·39 [1·76–3·25]). Results for *Shigella* spp, norovirus, and sapovirus suggested they had a stronger association with moderate-to-severe diarrhoea than other pathogens among children with better nutritional status, although *Shigella* spp remained associated with moderate-to-severe diarrhoea in both nutritional groups. 92 (64%) of 144 children with moderate-to-severe diarrhoea who died had acute malnutrition. Pathogen-specific 60-day fatality rates for all pathogens were higher among children with acute malnutrition, but no individual pathogen had a significantly larger increase in its relative association with mortality.

**Interpretation:**

Acute malnutrition might strengthen associations between specific pathogens and moderate-to-severe diarrhoea. However, the strong link between acute malnutrition and mortality during moderate-to-severe diarrhoea in children is not limited to specific infections, and affects a broad spectrum of enteric pathogens. Interventions addressing acute malnutrition could be an effective way to lower the mortality of both childhood malnutrition and diarrhoea.

**Funding:**

The Bill & Melinda Gates Foundation.

## Introduction

Children with acute malnutrition are three times more likely to die from diarrhoea than children with better nutritional status, and over 200 000 diarrhoeal deaths are attributed to acute malnutrition annually.^1^ The Global Enterics Multicenter Study (GEMS) established height-for-age Z-score as an important risk factor for mortality during episodes of moderate-to-severe diarrhoea,^2^ but the role of acute malnutrition (defined by a weight-for-height Z-score of ≤–2, presence of nutritional oedema, or a mid-upper arm circumference <12·5 cm) remains unclear. Acute malnutrition is associated with multiple physiological vulnerabilities, including immune dysfunction, enteric barrier disruption, gut microbiome dysbiosis, and essential nutrient deficits.^3^, ^4^, ^5^ Understanding how these vulnerabilities alter a child's risk of developing diarrhoea when exposed to a pathogen, and their risk of dying during a diarrhoeal episode, could inform interventions to reduce diarrhoeal disease mortality.

Enterotoxigenic *Escherichia coli*, enteroaggregative *E coli, Shigella* spp, *Salmonella* spp, *Campylobacter* spp, *Entamoeba histolytica,* and *Cryptosporidium* spp have been shown to be more common among children with acute malnutrition presenting with diarrhoea, compared with children who are better nourished.^6^, ^7^, ^8^, ^9^ Malnutrition-associated pathogens could be capitalising on the vulnerabilities of malnourished hosts. Alternatively, these differences in prevalence could be due to prolonged asymptomatic carriage among children with acute malnutrition. Acute malnutrition is also a risk factor for diarrhoea-associated mortality, but its effect on pathogen-specific mortality is unknown. If malnutrition strongly modifies a particular pathogen's association with death during diarrhoea, interventions targeting those specific pathogens among malnourished children could substantially reduce the mortality attributed to diarrhoea. However, if acute malnutrition uniformly increases mortality across diarrhoeal pathogens, children with acute malnutrition would benefit from pathogen-agnostic strategies to reduce rates of diarrhoeal mortality. Using GEMS data,^2^, ^10^, ^11^ we assessed whether acute malnutrition modifies the association between common enteric pathogens and moderate-to-severe diarrhoea. With ascertainment of vital status 60 days after enrolment, we further tested whether associations between enteric pathogens and death were modified by acute malnutrition among children with moderate-to-severe diarrhoea.

**Research in context**

**Evidence before this study**

Height-for-age Z-score, a marker of chronic malnutrition, was shown to be an important risk factor for death during episodes of childhood moderate-to-severe diarrhoea in the Global Enteric Multicenter Study (GEMS). However, little is known about acute malnutrition and its interactions with enteric pathogens. Previous data have suggested that members of the Enterobacteriacae family, *Campylobacter* spp, *Entamoeba histolytica*, and *Cryptosporidium* spp are more common among children with diarrhoea if that child has malnutrition, but these studies did not include community controls, limiting their ability to differentiate enteric pathogens associated with prolonged asymptomatic carriage from those that have an increased propensity to cause diarrhoea or death in children with acute malnutrition.

**Added value of this study**

Using cases of moderate-to-severe diarrhoea and community controls from GEMS, this analysis attempts to differentiate prolonged asymptomatic carriage of specific pathogens from increased virulence associated with malnutrition. We also compared the 60-day fatality rates of common enteric pathogens across childhood nutritional strata, to establish the contribution of individual pathogens to the high case fatality observed in children with acute malnutrition and moderate-to-severe diarrhoea.

**Implications of all the available evidence**

There is evidence that some pathogens are adept at capitalising on the vulnerabilities of children with acute malnutrition. However, the magnitude of these interactions does not explain the substantial increase in mortality observed in children with acute malnutrition. Children with moderate-to-severe diarrhoea who have acute malnutrition are at considerably higher risk of death than children with better nutritional status and diarrhoea, and children in the community with acute malnutrition. This increase in mortality occurs irrespective of the inciting pathogen, which suggests that pathogen-specific therapies will only lead to a modest reduction in malnutrition-associated diarrhoeal mortality. Interventions that address the broader scope of malnutrition-associated death could make an important contribution to reducing global diarrhoeal mortality.

## Methods

### Data collection

GEMS recruited children aged 0–59 months with moderate-to-severe diarrhoea and asymptomatic controls in Bangladesh, India, Kenya, Mali, Mozambique, Pakistan, and The Gambia.^2^, ^10^, ^11^, ^12^ Children were included if they presented to sentinel health centres between Dec 1, 2007, and March 3, 2011, with new (onset after ≥7 diarrhoea-free days) or acute (onset within the previous 7 days) diarrhoea, and with at least one of the following factors: sunken eyes, loss of skin turgor, intravenous rehydration required, dysentery, or admission to hospital. Cases were systematically sampled to represent the population presenting to the site, aiming to limit enrolment in each age stratum (<12, 12–23, and >24 months) to nine cases per fortnight at each site. One to three randomly selected controls without diarrhoea at home visits were matched to each diarrhoeal case. Matching was according to age (±2 months if the child was 0–23 months of age, ±4 months if the child was aged ≥24 months), sex, and community, and within 14 days of enrolment of the index case.

Detailed clinical, sociodemographic, and anthropometric data were collected at enrolment. Children received guideline-recommended treatment for diarrhoea at the discretion of the clinician, and cases and controls were visited approximately 60 days (range 50–90) after enrolment to measure vital status. Children with severe acute malnutrition were referred to local standard-of-care malnutrition management programmes, but some national guidelines do not include moderate acute malnutrition treatment.

All children provided at least 3 g of stool within 12 h of registration at the health facility, as previously described.^10^ If children required antibiotics before stool collection was possible, two rectal swabs for bacterial culture were taken before administration but a whole stool within 12 h of registration was still required for study inclusion. Bacterial culture techniques for stool and rectal swabs have been previously described.^10^, ^12^ Standard culture identified *E coli,* and *Salmonella, Shigella, Campylobacter, Aeromonas*, and *Vibrio* spp. Three *E coli* colonies were combined, and multiplex PCR was used to classify *E coli* pathotypes.^12^ Rotaviruses and adenoviruses were detected with the following immunoassays: ELISA ProSpecT Rotavirus kit (Oxoid, Basingstoke, UK), ProSpecT Adenovirus Microplate (Oxoid), and Premier Adenoclone kit, (Meridian Bioscience, Cincinnati, OH, USA). Multiplex reverse-transcriptase PCR detected norovirus and sapovirus. *Giardia lamblia, Entamoeba histolytica*, and *Cryptosporidium* spp were identified with commercially available enzyme-linked immunoassays (TechLab, Blacksburg, VA, USA).

### Statistical analysis

For this post-hoc analysis, acute malnutrition was defined as mid-upper arm circumference below 12·5 cm, capturing both severe acute malnutrition (<11·5 cm) and moderate acute malnutrition (≥11·5 cm and <12·5 cm). Mid-upper arm circumference is the optimal measure for identifying acute malnutrition among children with diarrhoea, as weight is more susceptible to dehydration.^13^, ^14^ Children with moderate-to-severe diarrhoea and matched controls were included in the analysis if their mid-upper arm circumference had been measured and if they were older than 6 months of age, as mid-upper arm circumference has not been validated in younger infants.

Conditional logistic regression was used to estimate the risk of diarrhoea given specific enteric infections in the age strata (0–11, 12–23, and 24–59 months), as per the GEMS primary analysis.^2^ Pathogens were modelled as present or absent, and to establish if they had a different association with diarrhoea among children with acute malnutrition, all models included an interaction term between the pathogen and acute malnutrition. When there was a consistent pattern of interaction across age strata (ie, all estimates were of a similar magnitude), interaction terms from a pooled age-group model were also reported. All models were adjusted for pathogens associated with moderate-to-severe diarrhoea in the original GEMS analysis (rotavirus, enterotoxigenic *E coli* producing heat-stable toxin [ST-ETEC], *Cryptosporidium* spp, *Shigella* spp*)* to control for confounding by co-infection. Controls were age-matched, but continuous and quadratic age in months were also tested in pooled age-group models. Interactions may differ between relative (odds ratio [OR]) and absolute (risk difference) models, therefore we also calculated relative excess risk due to interaction on the absolute risk scale.

The effect of acute malnutrition on the relationship between pathogens and mortality was assessed using 60-day follow-up data collected from moderate-to-severe diarrhoea cases. The incidence of death among moderate-to-severe diarrhoea cases with and without acute malnutrition was calculated using observed person-time and compared using a Kaplan-Meier plot and Cox proportional hazards regression. Children were censored at the date of death or at day 60 of follow-up. Children who died after 60 days post-enrolment were counted as alive at 60 days. Pathogen-specific mortality with 95% CIs were calculated separately by acute malnutrition status, assuming a Poisson distribution. The 60-day fatality rate for children in the control group with and without acute malnutrition was calculated as a reference for the main analysis. To test for interactions between acute malnutrition and specific pathogens, the hazard ratio (HR) for death associated with each pathogen, relative to children without that pathogen, among moderate-to-severe diarrhoea cases with and without acute malnutrition was calculated using Cox proportional hazards regression, and the interaction term between nutritional status and pathogen tested using the Wald statistic. To provide generalisability to current syndromic management, additional analyses were done to test an interaction between dysentery (present or absent) or acute watery diarrhoea (present or absent) and nutritional status. Dysentery was defined by the clinician or caregiver, or by laboratory-reported bloody stool. Acute watery diarrhoea was defined by the clinician when the child left the facility. There were too few deaths to stratify by age group, so Cox models were adjusted for age group as binary indicator variables and for pathogens associated with death in the original GEMS analysis (typical enteropathogenic *E coli* [EPEC], ST-ETEC, *Entamoeba histolytica, Cryptosporidium* spp), and clustered by site. Models including quadratic terms for age in months were run. All Cox models satisfied the proportional hazards assumption, which was tested using Schoenfeld residuals. Again, relative excess risk due to interaction models were used to calculate interactions on the absolute scale.

Although HIV status was not ascertained in GEMS, it could confound the relationships between enteric infections, acute malnutrition, and death. To understand whether HIV played a role in observed associations, we repeated mortality analyses in the subset of GEMS sites with low HIV prevalence (Bangladesh, India, Mali, Pakistan and The Gambia). Finally, to understand if socioeconomic status or height-for-age Z-score confounds the effect of malnutrition on diarrhoeal mortality, we further adjusted the Cox proportional hazards model for height-for-age Z-score, quadratic height-for-age Z-score, caregiver education (indicator variables: no school, less than primary, primary, secondary, post-secondary, religious education, unknown), household crowding (continuous), improved water source (binary), improved toilet type (binary), and a composite asset assessment constructed using a principal component analysis. The principal component methods are described in the appendix (p 3). All analyses were done in children with no missing data in STATA (version 14.2; StataCorp, College Station, TX, USA).

### Role of the funding source

The funder of the study had no role in in study design, data collection, data analysis, or data interpretation. An investigator employed by the funder (LML) participated in writing of the manuscript to provide expertise on diarrhoea aetiology and management.

## Results

**Figure 1:**
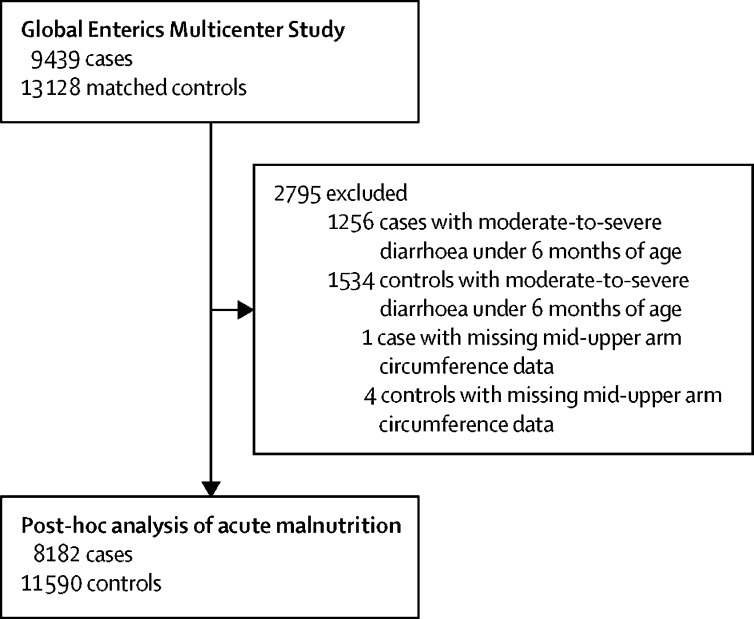
Flow chart of study inclusion

This post-hoc analysis included 8182 children aged 6–59 months with moderate-to-severe diarrhoea and 11 590 controls (figure 1). Children with acute malnutrition were younger than children with better nutritional status (table 1) and had a higher prevalence of severe dehydration and stunting, but were less likely to have dysentery. Only six children were affected by missing pathogens or covariate data. Among children with moderate-to-severe diarrhoea, 813 (9·9%) of 8182 had moderate acute malnutrition and 382 (4·7%) had severe acute malnutrition by mid-upper arm circumference (appendix p 1). 477 (4·1%) of 11 590 controls had moderate acute malnutrition, and 102 (0·9%) had severe acute malnutrition. Children with moderate-to-severe diarrhoea were three times more likely to be acutely malnourished (1195 [14·6%] of 8182) than age-matched and community-matched controls (579 [5·0%] of 11 590; p<0·0001). The percentage of cases and controls with acute malnutrition at sites with low HIV prevalence (943 [14·7%] of 6407 children with moderate-to-severe diarrhoea and 468 [5·3%] of 8916 controls) and at sites with high HIV prevalence (252 [14·2%] of 1775 children with moderate-to-severe diarrhoea and 111 [4·2%] of 2674 controls; appendix p 2) was similar. Children with acute malnutrition were more likely to be given antibiotics in the health centres (383 [32·1%] of 1195 children with acute malnutrition and moderate-to-severe diarrhoea *vs* 1442 [20·6%] of 6987 children with better nutritional status and moderate-to-severe diarrhoea), more likely to be admitted to hospital (337 [28·2%] children with acute malnutrition and moderate-to-severe diarrhoea *vs* 1285 [18·4%] children with better nutritional status and moderate-to-severe diarrhoea), and less likely to be prescribed outpatient antibiotics (664 [55·6%] children with acute malnutrition and moderate-to-severe diarrhoea *vs* 5332 [76·3%] children with better nutritional status and moderate-to-severe diarrhoea) than children with better nutritional status.

**Table 1:** Baseline characteristics of the study population

		**Acute malnutrition (n=1774)**	**Better nutritional status (n=17 998)**
Age (months)			
	6–11	897 (50·6%)	5216 (29·0%)
	12–23	723 (40·8%)	6863 (38·1%)
	24–59	154 (8·7%)	5919 (32·9%)
Sex			
	Female	961 (54·2%)	7587 (42·2%)
	Male	213 (45·8%)	10 411
Breastfeeding			
	Exclusively	78 (4·4%)	613 (3·4%)
	In addition to complementary foods	1341 (75·6%)	11 750 (65·3%)
Stunted growth (length-for-age Z-score ≤2)		1055 (59·5%)	4819 (26·8%)
Dysentery in past 7 days		150 (8·5%)	1771 (9·8%)
Dehydration			
	Some	807 (45·5%)	4375 (24·3%)
	Severe	186 (10·5	630 (3·5%)
Rectal swab		25 (1·4%)	83 (<1%)
Site			
	Bangladesh	156 (8·8%)	3320 (18·4%)
	India	200 (11·3%)	2943 (16·4%)
	Kenya	227 (12·8%)	2621 (14·6%)
	Mali	317 (17·9%)	3395 (18·9%)
	Mozambique	136 (7·7%)	1465 (8·1%)
	Pakistan	482 (27·2%)	2074 (11·5%)
	The Gambia	256 (14·4%)	2180 (12·1%)
Biological mother is primary caregiver		1711 (96·4%)	17 303 (96·1%)
Primary caregiver completed primary school		649 (36·6%)	9001 (50·0%)
Improved main water source*		1435 (80·9%)	15 488 (86·1%)
Treated water		511 (28·8%)	4240 (23·6%)
Improved toilet facilities†		679 (38·3%)	7077 (39·3%)

Data are n (%). Children had acute malnutrition with a mid-upper arm circumference below 12·5 cm, and had better nutritional status with a mid-upper arm circumference of at least 12·5 cm.

* Improved water sources were piped water, public taps, tube wells, rainwater, covered wells, protected springs, and bore holes. Unimproved water sources were open wells, surface water, unprotected springs, bought water.

† Improved toilet facilities were flushing toilets, improved pit latrines, and pour flush toilets. Unimproved toilet facilities were traditional pit latrines and no facilities.

At least one enteric pathogen was identified in 6726 (82·2%) of 8182 cases and 8198 (70·7%) of 11 590 controls. In adjusted and unadjusted models (appendix p 4), acute malnutrition modified age-specific odds of diarrhoea for typical EPEC among children aged 6–11 months, ST-ETEC among children aged 12–23 months, *Shigella* spp among children aged 12–23 months and 24–59 months, and sapovirus among children aged 24–59 months (table 2). The age, site, and co-infection adjusted odds ratios (aORs) for moderate-to-severe diarrhoea associated with EPEC among children aged 6–11 months was 2·08 (95% CI 1·14–3·79) in children with acute malnutrition, and 0·97 (0·77–1·23) in children with better nutritional status, compared with healthy controls. ST-ETEC among children aged 12–23 months also had a stronger association with moderate-to-severe diarrhoea in children with acute malnutrition (aOR 7·60 [2·63–21·95]) than among similarly aged children with better nutritional status (aOR 2·39 [1·76–3·25]). Additionally, there was some evidence of interaction for ST-ETEC, *Shigella* spp, and norovirus among children aged 6–11 months, and norovirus, *Vibrio* spp, and typical EPEC among children aged 12–24 months. The typical EPEC and moderate-to-severe diarrhoea association was 2-fold stronger among children with acute malnutrition than among those with better nutritional status, with a pooled age-group ratio of adjusted OR (rOR) of 2·09 (95% CI 1·33–3·30). Similarly, ST-ETEC had a stronger association with moderate-to-severe diarrhoea in children with acute malnutrition across all age groups (rOR 2·59 [1·18–5·71]). Conversely, *Shigella* spp had a consistently stronger association with moderate-to-severe diarrhoea among children without malnutrition in each age strata, with a pooled age-group rOR of 0·28 (0·15–0·53). The association of norovirus with moderate-to-severe diarrhoea had an inverse interaction with nutritional status across age strata; norovirus had a 28% weaker association with moderate-to-severe diarrhoea among children with acute malnutrition than among children with better nutritional status (rOR 0·72 [0·46–1·12]). The pooled age-group rOR for sapovirus was 0·55 (0·30–0·99). Rotavirus appeared to have no interaction with malnutrition in relative models, but in absolute models there was a higher risk of moderate-to-severe diarrhoea when rotavirus was detected among acutely malnourished compared with better nourished children (appendix p 5). There also was no evidence of an interaction with ETEC in the additive models, but all other results of relative and absolute models were concordant. No additional control for confounding was provided by inclusions of age as a quadratic term in any of the age-group pooled models.

**Table 2:** Associations with diarrhoea for the pathogens with the largest attributable fractions in the Global Enteric Multicenter Study, stratified by nutritional status displayed in each age stratum

	**Acute malnutrition**			**Better nutritional status**		
	Diarrhoea cases (n=1195)	Controls (n=579)	Adjusted OR* (95% CI)	Diarrhoea cases (n=6987)	Controls (n=11 011)	Adjusted OR* (95% CI)
**6–11 months (n=6113)**						
*Shigella* spp	24 (3·9%)	1 (<1%)	14·90 (1·76–126·43)	127 (5·9%)	11 (<1%)	25·09 (12·59–50·03)
Rotavirus	170 (28·0%)	10 (3·5%)	12·88 (6·22–26·68)	557 (26·0%)	155 (5·1%)	11·98 (9·06–15·85)
Adenovirus 40 and 41	20 (3·3%)	3 (1·0%)	11·30 (2·41–52·99)	67 (3·1%)	31 (1·0%)	5·22 (3·08–8·85)
ST-ETEC	35 (5·8%)	4 (1·4%)	5·86 (1·95–17·59)	99 (4·6%)	57 (1·9%)	3·47 (2·30–5·25)
*Cryptosporidium* spp	122 (20·1%)	29 (10·0%)	3·23 (1·90–5·48)	348 (16·0%)	217 (7·1%)	3·18 (2·53–4·00)
*Vibrio* spp	10 (1·6%)	1 (<1%)	8·13 (1·01–65·62)	22 (1·0%)	17 (<1%)	1·96 (0·91–4·21)
*Entamoeba* spp	17 (2·8%)	4 (1·4%)	2·05 (0·51–8·28)	76 (3·5%)	67 (2·2%)	1·89 (1·19–3·01)
Norovirus	39 (6·4%)	27 (9·3%)	1·07 (0·56–2·04)	205 (9·5%)	224 (7·3%)	1·53 (1·20–1·95)
*Aeromonas* spp	39 (6·4%)	15 (5·2%)	2·64 (1·17–5·95)	131 (6·1%)	129 (4·2%)	1·51 (1·07–2·14)
*Campylobacter jejuni*	58 (9·5%)	35 (12·0%)	1·07 (0·62–1·85)	283 (13·0%)	297 (9·7%)	1·48 (1·19–1·85)
EPEC	71 (11·7%)	24 (8·3%)	2·08 (1·14–3·79)†	193 (8·9%)	323 (10·6%)	0·97 (0·77–1·23)†
**12–23 months (n=7586)**						
*Shigella* spp	52 (10·8%)	9 (3·7%)	3·21 (1·50–6·88)†	432 (16·0%)	96 (2·3%)	10·81 (8·33–14·03)†
Rotavirus	77 (16·0%)	10 (4·1%)	4·19 (2·05–8·56)	495 (18·0%)	163 (3·9%)	8·17 (6·50–10·27)
Adenovirus 40 and 41	10 (2·1%)	5 (2·1%)	1·20 (0·36–4·04)	79 (2·9%)	37 (<1%)	3·57 (2·30–5·53)
ST-ETEC	37 (7·7%)	5 (2·1%)	7·60 (2·63–21·95)†	133 (4·9%)	97 (2·3%)	2·39 (1·76–3·25)†
*Cryptosporidium* spp	86 (17·9%)	23 (9·5%)	2·57 (1·47–4·49)	288 (11·0%)	253 (6·1%)	2·09 (1·69–2·59)
*Vibrio* spp	15 (3·1%)	4 (1·7%)	2·40 (0·70–8·23)	81 (3·0%)	19 (<1%)	9·09 (5·00–16·53)
*Entamoeba* spp	15 (3·1%)	9 (3·7%)	0·94 (0·36–2·44)	85 (3·1%)	89 (2·2%)	1·57 (1·05–2·34)
*Aeromonas* spp	34 (7·1%)	12 (5·0%)	2·11 (0·94–4·74)	180 (6·6%)	205 (5·0%)	2·04 (1·53–2·73)
Salmonella (non-typhoidal)	12 (2·5%)	8 (3·3%)	1·02 (0·36–2·89)	45 (1·7%)	54 (1·3%)	1·86 (1·17–2·96)
**24–59 months (n=6073)**						
*Shigella* spp	16 (15·1%)	4 (8·3%)	2·23 (0·64–7·83)†	409 (19·0%)	90 (2·4%)	15·11 (11·37–20·08)†
Rotavirus	11 (10·4%)	0	..	148 (7·1%)	91 (2·4%)	4·13 (3·01–5·66)
ST-ETEC	7 (6·6%)	0	..	81 (3·9%)	64 (1·7%)	2·48 (1·70–3·60)
*Vibrio* spp	9 (8·5%)	0	..	92 (4·4%)	23 (<1%)	16·00 (9·13–28·05)
*Entamoeba* spp	4 (3·8%)	1 (2·1%)	2·94 (0·30–28·55)	60 (2·9%)	68 (1·8%)	1·69 (1·09–2·63)
*Aeromonas* spp	11 (10·4%)	4 (8·3%)	2·90 (0·69–12·16)	179 (8·5%)	206 (5·4%)	2·75 (2·05–3·69)
*Campylobactor jejuni*	15 (14·2%)	7 (15·0%)	1·41 (0·46–4·29)	154 (7·3%)	263 (6·9%)	1·51 (1·17–1·96)
Salmonella (non-typhoidal)	5 (4·7%)	0	..	41 (2·0%)	29 (<1%)	3·02 (1·78–5·11)
Sapovirus	1 (<1%)	4 (8·3%)	0·03 (0·00–0·45)†	54 (2·6%)	101 (2·6%)	1·01 (0·69–1·49)†

Unadjusted estimates are available in the appendix (p 4). OR=odds ratio. ST-ETEC=enterotoxigenic *Escherichia coli* producing heat-stable toxin. EPEC=enteropathogenic *E coli*.

* Adjusted for pathogens associated with diarrhoea (ST-ETEC, *Shigella* spp, *Cryptosporidium* spp, rotavirus).

† Significant (p<0·05) interaction between acute malnutrition status and pathogen in this age strata.

**Figure 2:**
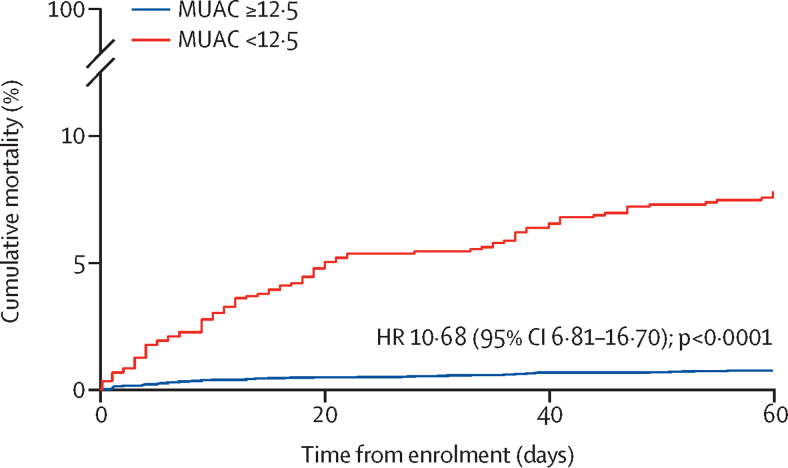
Cumulative mortality of children with moderate-to-severe diarrhoea stratified by nutritional status 604 (8·6%) of 6987 children with MUAC ≥12·5 cm and 112 (9·4%) of 1195 children with MUAC <12·5 cm had no recorded ascertainment of vital status during follow-up. The HR displayed in the figure is the crude HR at day 60. The HR was 9·99 (95% CI 5·70–17·53; p<0·0001) at day 20 and 9·52 (5·50–16·48; p<0·0001) at day 40. MUAC=mid-upper arm circumference. HR=hazard ratio.

Of 172 deaths in the study population, 144 (83·7%) were among children with moderate-to-severe diarrhoea. 92 (64%) of 144 children with moderate-to-severe diarrhoea who died had acute malnutrition (36 [25%] had moderate acute malnutrition, 56 [39%] had severe acute malnutrition). The 60-day fatality rate (case fatality rate) for children with acute malnutrition and moderate-to-severe diarrhoea was 8·2 deaths per 100 children (95% CI 6·7–10·1), with a median time to death of 15 days (IQR 5–35; figure 2). Among children who had moderate-to-severe diarrhoea without acute malnutrition, the case fatality rate was 0·8 deaths per 100 children (0·6–1·0) and the median time to death was 9·5 days (4–32). Among children without moderate-to-severe diarrhoea, the case fatality rate for those with acute malnutrition was 0·9 deaths per 100 children (0·4–2·1) and for those without malnutrition it was 0·2 deaths per 100 children (0·1–0·3). The crude HRs comparing children with and without acute malnutrition were 10·68 (95% CI 6·81–16·70; p<0·0001) among children with moderate-to-severe diarrhoea, and 4·31 (1·60–11·70; p=0·004) among controls. Adjustment for socioeconomic status and height-for-age Z-score led to a small reduction in the magnitude of association between acute malnutrition and mortality among children with moderate-to-severe diarrhoea (adjusted HR 7·38, 95% CI 4·83–11·28). Acute malnutrition had a similar association with death both at sites with high HIV prevalence and at sites with low HIV prevalence (table 3).

**Table 3:** Associations between acute malnutrition and deaths among children with moderate-to-severe diarrhoea

	**Deaths**	**Person time observed (days)**	60-day fatality rate*(95% CI)	**Hazard ratio**
**All sites**				
Better nutritional status (n=6383)	52 (<1%)	408 920	0·8 (0·6–1·0)	1 (ref)
Acute malnutrition (n=1071)	92 (8·6%)	66 945	8·2 (6·7–10·1)	10·7 (6·8–16·7)
**Sites with low HIV prevalence**				
Better nutritional status (n=4934)	22 (<1%)	325 224	0·4 (0·3–0·6)	1 (ref)
Acute malnutrition (n=834)	42 (5·0%)	54 632	4·6 (3·4–6·2)	11·3 (6·7–18·9)

Children had acute malnutrition with a mid-upper arm circumference below 12·5 cm, and had better nutritional status with a mid-upper arm circumference of at least 12·5 cm.

* Incidence of mortality was calculated using observed person-time and aggregated to give 60-day fatality per 100 children.

Each pathogen-specific case fatality rate was higher among children with acute malnutrition than among children with better nutritional status (table 4). The five pathogens with the largest malnutrition-associated increases in case fatality were *Entamoeba histolytica*, typical EPEC, *Shigella* spp, atypical EPEC, and ST-ETEC, although these interaction terms were not significant. Cox regression models comparing children with and without detectable *Cryptosporidium* spp found this pathogen to have a 33% weaker association with mortality among children with acute malnutrition (ratio of hazard ratios [rHR] 0·67 [95% CI 0·58–0·78]; table 5), compared with the same association among children with better nutritional status. Similarly, norovirus had a weaker association with mortality among children with acute malnutrition (rHR 0·24 [0·08–0·77]) than among children with better nutritional status. Rotavirus (rHR 0·49 [0·17–1·39]) and sapovirus (rHR 0·57 [0·11–2·90]) estimates also suggest an interaction with nutritional status. Acute watery diarrhoea interacted with nutritional status (rHR 0·37 [0·18–0·74]), suggesting the association between a final diagnosis of watery diarrhoea and mortality was 63% weaker among children with acute malnutrition than the same comparison among children with better nutritional status. Conversely, dysentery had a 2·6-fold stronger association with mortality in children with acute malnutrition (rHR 2·63 [0·83–8·46]) than in children with better nutritional status. Finally, *Shigella* spp showed some evidence of effect modification by nutritional status (rHR 3·13 [0·82–11·91]), but the remaining pathogens either showed no evidence of an interaction, or were too rare for an accurate estimation.

**Table 4:** Pathogen-specific 60-day crude fatality rates among children with moderate-to-severe diarrhoea stratified by nutritional status

	**Acute malnutrition (n=1071)**			**Better nutritional status (n=6383)**		
	N	Deaths (n=92)	Crude 60-day fatality rate* (95% CI)	N	Deaths (n=52)	Crude 60-day fatality rate* (95% CI)
**Viruses**						
Adenovirus	27	2/27	6·8 (1·7–27·2)	154	1/154	0·6 (0·1–4·3)
Norovirus	73	2/73	2·5 (0·6–10·0)	512	5/513	0·9 (0·4–2·2)
Rotavirus	235	12/235	4·9 (2·8–8·6)	1088	10/1088	0·9 (0·5–1·6)
Sapovirus	36	2/36	5·0 (1·2–19·9)	226	2/226	0·8 (0·2–3·2)
**Bacteria**						
*Aeromonas* spp	71	4/71	4·9 (1·8–13·1)	448	0	..
*Campylobacter jejuni*	116	3/116	2·3 (0·7–7·0)	616	2/616	0·3 (0·1–1·2)
*Vibrio* spp	30	1/30	3·0 (0·4–21·6)	177	2/177	1·0 (0·3–4·2)
EAEC	236	25/236	10·4 (7·0–15·4)	1128	11/1128	0·9 (0·5–1·6)
EHEC	1	0	..	2	0	..
Atypical EPEC	35	4/35	11·2 (4·2–29·8)	233	1/233	0·4 (0·6–2·9)
Typical EPEC	115	18/115	15·8 (10·0–25·1)	451	6/451	1·2 (0·5–2·7)
ETEC producing heat-stable toxin	66	8/66	11·1 (5·6–22·3)	280	3/280	0·9 (0·3–3·1)
ETEC producing heat-labile toxin	51	3/51	5·4 (1·7–16·8)	294	1/294	0·3 (0·4–2·2)
Salmonella (non-typhoidal)	29	3/29	10·6 (3·4–33·8)	128	4/128	3·2 (1·2–8·5)
*Shigella* spp	89	10/89	12·0 (6·5–22·3)	923	4/923	0·4 (0·2–1·1)
**Protozoa**						
*Cryptosporidium* spp	196	22/196	11·0 (7·3–16·7)	686	11/686	1·5 (0·8–2·7)
*Entamoeba* spp	36	6/36	19·4 (8·7–43·2)	207	4/207	1·9 (0·7–5·0)
*Giardia lamblia*	155	8/155	4·7 (2·3–9·3)	1402	5/1402	0·3 (0·1%-0·7)

No tests of significance were done for the crude fatality rates in this table. EAEC=enteroaggregative *Escherichia coli*. EHEC=enterohaemorrhagic *E coli*. EPEC=enteropathogenic *E coli*. ETEC=enterotoxigenic *E coli*.

* Incidence of mortality was calculated using observed person-time and aggregated to give 60-day fatality per 100 children.

**Table 5:** Pathogen-specific HRs for mortality among children with moderate-to-severe diarrhoea stratified by nutritional status

		**Acute malnutrition**		**Better nutritional status**		**Interaction term (ratio of HRs)**
		Crude HR*	Adjusted HR†	Crude HR*	Adjusted HR†	
Viruses						
	Adenovirus	0·84 (0·25–2·86)	0·74 (0·21–2·61)	0·79 (0·11–5·53)	0·81 (0·11–5·90)	0·92 (0·15–5·71)
	Norovirus	0·29 (0·10–0·89)‡	0·30 (0·10–0·92)‡	1·22 (0·45–3·27)‡	1·23 (0·45–3·34)‡	0·24 (0·08–0·77)
	Rotavirus	0·54 (0·24–1·19)	0·56 (0·24–1·30)	1·15 (0·48–2·77)	1·15 (0·50–2·65)	0·49 (0·17–1·39)
	Sapovirus	0·60 (0·19–1·92)	0·60 (0·17–2·14)	1·06 (0·27–4·19)	1·05 (0·26–4·20)	0·57 (0·11–2·90)
Bacteria						
	*Aeromonas* spp	0·59 (0·23–1·53)	0·64 (0·25–1·64)	..	..	..
	*Campylobacter jejuni*	0·25 (0·12–0·53)	0·27 (0·13–0·58)	0·37 (0·14–1·00)	0·37 (0·13–1·02)	0·73 (0·18–3·01)
	*Vibrio* spp	0·37 (0·04–3·39)	0·35 (0·04–3·08)	1·38 (0·35–5·41)	1·46 (0·39–5·49)	0·24 (0·01–5·10)
EAEC		1·36 (0·86–2·15)	1·45 (0·83–2·54)	1·22 (0·52–2·89)	1·29 (0·54–3·11)	1·13 (0·58–2·18)
Atypical EPEC		1·35 (0·88–2·05)	1·26 (0·80–1·96)	0·52 (0·10–2·85)	0·53 (0·10–3·00)	2·35 (0·36–15·30)
Typical EPEC		2·11 (1·44–3·07)	2·18 (1·46–3·28)	1·69 (0·79–3·59)	1·72 (0·80–3·69)	1·27 (0·64–2·50)
	ETEC producing heat-stable toxin	1·37 (0·74–2·54)	1·52 (0·75–3·09)	1·31 (0·41–4·14)	1·39 (0·44–4·32)	1·09 (0·20–5·96)
	ETEC producing heat-labile toxin	0·65 (0·40–1·05)	0·71 (0·39–1·26)	0·40 (0·08–2·14)	0·41 (0·08–2·15)	1·70 (0·40–7·27)
Salmonella (non-typhoidal)		1·26 (0·56–2·83)	1·31 (0·60–2·85)	4·36 (0·99–19·17)	4·55 (1·01–20·32)	0·29 (0·05–1·66)
*Shigella* spp		1·51 (0·69–3·28)	1·57 (0·75–3·31)	0·52 (0·09–2·99)	0·50 (0·08–3·04)	3·13 (0·82–11·91)
Protozoa						
	*Cryptosporidium* spp	1·43 (0·80–2·56)‡	1·48 (0·82–2·68)‡	2·22 (1·00–4·93)‡	2·20 (1·06–4·55)‡	0·67 (0·58–0·78)
	*Entamoeba* spp	2·41 (1·17–4·96)	2·58 (1·21–5·50)	2·57 (0·66–9·97)	2·65 (0·69–10·2)	0·97 (0·35–2·72)
	*Giardia lamblia*	0·53 (0·21–1·31)	0·55 (0·20–1·51)	0·37 (0·13–1·05)	0·36 (0·13–1·04)	1·50 (0·45–4·98)

HR=hazard ratio. EAEC=enteroaggregative *Escherichia coli*. EPEC=enteropathogenic *E coli*. ETEC=enterotoxigenic *E coli*.

* Clustered by site.

† Adjusted for age and pathogens associated with death during diarrhoea in the original Global Enterics Multicenter Study (*Cryptosporidium* spp, *Entamoeba* spp, ST-ETEC, and typical EPEC) and clustered by site.

‡ Significant (p<0·05) interaction between pathogen or group of pathogens and acute malnutrition.

In absolute models, *Cryptosporidium* spp, *Entamoeba* spp, and typical EPEC trended toward having an interaction with nutritional status that suggested these pathogens were associated with additional risk of death among children with acute malnutrition (appendix p 6). ETEC producing heat-labile toxin appeared to have an inverse interaction with nutritional status in absolute models. All other interactions appeared to be similar between the relative and absolute scales. No additional control for confounding was provided by inclusions of age as a quadratic term in any of the Cox models.

## Discussion

This post-hoc, exploratory analysis found typical EPEC and ST-ETEC to have a stronger association with moderate-to-severe diarrhoea among children with acute malnutrition than among children with better nutritional status. Conversely, *Shigella* spp*,* norovirus, and sapovirus had a more pronounced association with moderate-to-severe diarrhoea in children with better nutritional status. Children with acute malnutrition were at high risk of mortality following an episode of moderate-to-severe diarrhoea and represented nearly two-thirds of the deaths observed among children with moderate-to-severe diarrhoea. The case fatality for every pathogen was higher among children with acute malnutrition, but this increase was less pronounced among children with norovirus, *Cryptosporidium* spp, or acute watery diarrhoea.

The GEMS control group enabled this analysis to differentiate malnutrition-associated differences in diarrhoea aetiology from pathogens that are more prevalent among malnourished children due to prolonged carriage.^15^, ^16^ Previous evidence suggests that malnourished children are particularly vulnerable to pathogenic *E coli*,^17^, ^18^, ^19^ and we found ST-ETEC and typical EPEC to have a stronger association with diarrhoea among children with acute malnutrition. Many of the most virulent pathogens (*Shigella* spp, rotavirus, and *Cryptosporidium* spp*)* had contradictory results in relative and absolute risk models. For example, in logistic regression *Shigella* spp had a stronger association with moderate-to-severe diarrhoea among children without acute malnutrition, but this interaction was not present when looking at absolute risk. Because particularly virulent pathogens can cause severe disease in otherwise healthy children whereas less virulent pathogens may only cause severe disease in children with underlying vulnerabilities, the relative importance of virulent pathogens may appear greatest among children with better nutritional status.

Linear growth stunting was an important risk factor for mortality in the original GEMS analysis.^2^ Acute malnutrition also appears to be an important risk factor for death during and after moderate-to-severe diarrhoea, independent of height-for-age Z-score. Children with moderate-to-severe diarrhoea who had acute malnutrition were at substantially higher risk of mortality than children with acute malnutrition in the control group, suggesting that the combination of acute malnutrition and diarrhoea identifies children at particularly high risk of death. Approximately a quarter of the deaths observed in this analysis were among children with moderate acute malnutrition, a group for which there is no specific management guideline, but who could be an important target population to reduce child mortality.

Enteric dysfunction and microbiome dysbiosis are linked to acute malnutrition,^20^, ^21^ and might allow enteric pathogens to more readily cause fatal disease. We found some evidence of a stronger association between dysentery and death among children with acute malnutrition than among children with better nutritional status. A recent study in Mozambique^22^ noted dysentery to have an inverse association with death, and suggested that this could be linked to the treatment of dysentery with antibiotics. Antibiotic effectiveness among malnourished children might be decreased by delayed presentation to hospital, increased prevalence of antibiotic-resistant organisms,^23^ or an altered pharmacokinetic profile,^24^ which could explain the stronger association between dysentery and death in this group. Additionally, children with acute malnutrition are thought to be particularly vulnerable to dehydration, fluid overload, and electrolyte imbalances.^25^ Interestingly, the increase in mortality associated with malnutrition in this analysis was smallest among children with acute watery diarrhoea, perhaps due to the fact that GEMS ensured guidelines for diarrhoea treatment were implemented across the cohort. A lower than expected mortality among children with acute malnutrition and viral pathogens, and among children with acute malnutrition and acute watery diarrhoea, might indicate that current diarrhoea management guidelines are more effective at preventing death due to dehydration than at preventing other pathways to mortality.

Malnourished children are more likely to present with Gram-negative sepsis than children who are better nourished,^17^, ^18^, ^26^ and we found four of the five pathogens with the largest malnutrition-associated increases in mortality were Gram-negative Enterobacteriaceae. EPEC, ST-ETEC, and *Shigella* spp are not commonly associated with sepsis or bacteraemia, but infection with these pathogens could indirectly facilitate Gram-negative sepsis. A study in Kenya,^27^ in which children with severe acute malnutrition were twice as likely to die if they had diarrhoea at admission, found that among malnourished children with diarrhoea, Gram-negative organisms were present in 53% of blood culture isolates at admission and in 83% of isolates from malnourished children with hospital-acquired diarrhoea. The combined risk of diarrhoea, acute malnutrition, and Gram-negative sepsis reinforces the need to consider antibiotics for children with moderate-to-severe diarrhoea and moderate acute malnutrition, and to evaluate additional treatment regimens for children with severe acute malnutrition. The Antibiotics for Children with severe Diarrhea trial (registered with ClinicalTrials.gov, NCT03130114) and the First Line Antimicrobials in Complicated Severe Acute Malnutrition trial (registered with ClinicalTrials.gov, NCT03174236) will offer important insights into antibiotic regimens for malnourished children.

We found scarce evidence of confounding by socioeconomic status, but capturing socioeconomic status across diverse settings is challenging, and the data could be subject to social desirability bias, suggesting socioeconomic vulnerability could still explain some of the relationship between malnutrition and mortality. Pathogenic bacteria could be a marker of socioeconomic status through inadequate access to water, sanitation, and hygiene facilities, and mid-upper arm circumference is also a measure of socioeconomic vulnerability. Children with both low mid-upper arm circumference and enteric bacterial pathogens could come from more disadvantaged social situations. Incorporating a holistic assessment of childhood vulnerability into management could provide a more accurate method of targeting interventions, such as an increased frequency of follow-up appointments and young infant feeding interventions.

This analysis has several important limitations. HIV is strongly associated with acute malnutrition, certain enteric pathogens, and death, and HIV exposure or infection can confound some of these results. However, analyses at sites with a low HIV prevalence suggest that this confounding is minimal. Misclassification of nutritional status can also affect our results, as dehydration does reduce mid-upper arm circumference and is a strong risk factor for death. However, mid-upper arm circumference is the most robust measure of nutritional status among children with diarrhoea.^13^, ^14^ Additionally, GEMS excluded children with mild diarrhoea, which prevents us from drawing conclusions regarding the full spectrum of diarrhoeal disease. The case selection strategy was limited to nine children per week, and so without including sampling weights, cases are not representative of all children who would meet the inclusion criteria. The generalisability of our findings may also be limited to context with a similar adherence to WHO rehydration recommendations, a comparable prevalence of antibiotic use, and similar malnutrition management. As an exploratory analysis, we ran a large number of hypothesis tests which could have led to type I error. Finally, re-analysis of stool samples using molecular techniques revealed that traditional microbiological methods underestimate the prevalence of many pathogens, particularly *Shigella* spp.^28^ If this misclassification was differential across nutritional strata, we would expect substantially different results using the molecular data.

Acute malnutrition is a crucial predictor of death during and after an episode of moderate-to-severe diarrhoea, and clinicians should be aware that children with acute malnutrition and diarrhoea are at high risk for mortality and consider additional follow-up and close monitoring. There is a clear need for improved evidence to guide the management of children with moderate acute malnutrition, who comprise a large population at high risk for mortality, for whom no widely implemented management strategies have been defined. The increased mortality associated with malnutrition, irrespective of the infecting pathogen, suggests that interventions targeting specific pathogens would only lead to modest reductions in malnutrition-associated diarrhoeal mortality, and that more lives could be saved through interventions that address underlying malnutrition and the mechanistic pathways that contribute to malnutrition-associated vulnerability.
