# Supplementary material for: The effect of acute malnutrition on enteric pathogens, moderate-to-severe diarrhoea, and associated mortality in the Global Enteric Multicenter Study cohort: a post-hoc analysis
Source: Lancet Glob Health. 2020 Jan 22;8(2):e215–24. doi: 10.1016/S2214-109X(19)30498-X (PMC7025322; doi:10.1016/S2214-109X(19)30498-X)
Supplement: Supplementary appendix [file mmc1.pdf]

THE LANCET
Global Health

Supplementary appendix

This appendix formed part of the original submission and has been peer reviewed. We post it as supplied by the authors.

Supplement to: Tickell KD, Sharmin R, Deichsel EL, et al. The effect of acute malnutrition on enteric pathogens, moderate-to-severe diarrhoea, and associated mortality in the Global Enteric Multicenter Study cohort: a post-hoc analysis. *Lancet Glob Health* 2020; **8:** e215–24.

Supplementary Appendix 1: **Baseline characteristics of included cases and controls.**

	**Cases** N: 8,182		**Controls** N: 11,590	
	n	%	n	%
**Child Characteristics**				
Age				
6-11 months	2,772	34%	3,341	29%
12-23 months	3,205	39%	4,381	38%
24-59 months	2,205	27%	3,868	33%
Sex (female)	3,551	43%	4,997	43%
Currently exclusively breast feeding	239	3%	452	4%
Currently any breast feeding	5,545	68%	7,547	65%
Underweight[1]	2,670	33%	2,574	22%
Stunted (LAZ <-2)	2,386	29%	3,488	30%
MAM[2]	813	10%	477	4%
SAM[2]	382	5%	102	1%
Dysentery in last 7 days	1,922	23%	82	1%
Rectal swab	108	1%	0	0%
Site				
Bangladesh	1,250	15%	2,226	19%
India	1,349	16%	1,794	15%
Kenya	1,230	15%	1,618	14%
Mali	1,854	23%	1,858	16%
Mozambique	545	7%	1,056	9%
Pakistan	991	12%	1,565	14%
The Gambia	963	12%	1,473	13%
**Social & economic status**				
Biological mother is primary caregiver	7,823	96%	11,191	97%
Primary caregiver completed primary school	3,826	47%	5,832	50%
Improved main water source[3]	6,882	87%	10,042	89%
Treats water	2,278	28%	2,473	21%
Improved toilet[4]	3,099	38%	4,657	40%

Abbreviations: LAZ: Length-for-age z-score, MAM: moderate acute malnutrition, SAM: Severe acute malnutrition.

[1]Weight-for-height z-score based on post-rehydration weight when rehydration required.

[2]Defined by MUAC alone (MAM <12·5cm & ≥ 11·5cm, SAM <11·5cm). [3]Improved water sources: piped water, public tap, tube well, rainwater, covered well, protected spring, bore hole. Unimproved water source: open wells, surface water, unprotected spring, bought water.

[4]Improved toilet: flush toilet, improved pit latrine, pour flush toilet. Unimproved: traditional pit latrine, no facility.

<u>Supplementary Appendix 2:</u> Diarrhea cases and controls by malnutrition status in low-HIV prevalence sites.

	**Low-HIV Prevalence Sites**		**High HIV Prevalence Sites**	
	**Acute malnutrition N (%)**	**No acute malnutrition N(%)**	**Acute malnutrition N (%)**	**No acute malnutrition N(%)**
Diarrhea Case	943 (15%)	5,464 (85%)	252 (14%)	1,523 (86%)
Control	468 (6%)	8,448 (95%)	111 (4%)	2,563 (96%)
**Total**	**1,411**	**13,912**	**363**	**7,481**

<u>Supplementary Appendix 3:</u>
Household asset assessment: The below list of binary variables were included in a principle component analysis, and the optimal number of components was chosen using a Scree plot. A further sensitivity analysis using all components with eigenvalues greater than or equal to 1 was also conducted. Only eight children had missing socio-economic data, so a complete case approach was taken. The crude cox-proportional hazard model was limited to children with a full complement of socioeconomic variables to ensure an accurate comparison was made.

Asset variables: Household flooring (indicator variables for earth/sand, dung, wood planks, palm/bamboo, polished wood, vinyl or asphalt, ceramic tile, cement, carpet), Household electricity, television, motorcycle, radio, bike, car, fridge, boat, telephone, cart, agricultural land.

Results: The crude hazard rate in this analysis was 10·68 (95% CI: 6·81-16·70). The Scree plot (Figure 1) suggested that four principle components was the optimal number to include in models. As noted in the manuscript inclusion of these components and the other markers of socioeconomic was not consistent with confounding (adjusted HR: 10.16, 95% CI: 6·70-15·43). However, the four selected components only captured 41% of the variability in household assets. Using the nine components with eigenvalues greater than or equal to one, we were able to capture 65% of the asset variability, but inclusion of these components did not further effect the estimated relationship between acute malnutrition and death (HR: 10.16, 95% CI: 6.64-15.52).

Supplementary Figure 1: A Scree plot of eigenvalues derived the principle component analysis of household assets

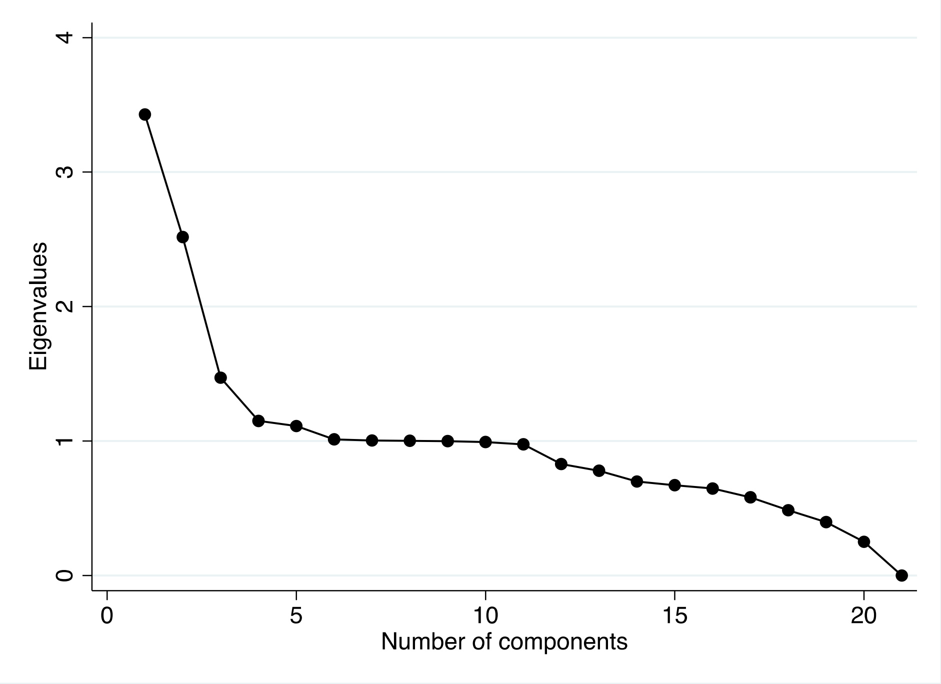

Supplementary Appendix 4: Unadjusted estimates for the association with diarrhea for the pathogens with the largest attributable fractions in the Global Enteric Multicenter study stratified by acute nutritional status displayed in each age stratum.

	Acute Malnutrition (1195 cases, 579 controls)					No Acute Malnutrition (6987 cases, 11011 controls)				
	Diarrhea Cases		Controls		OR of Diarrhea	Diarrhea Cases		Controls		OR of Diarrhea
Infection	N	%	N	%	OR (95% CI)[1]	N	%	N	%	OR (95% CI)[1]
6-11 months										
Shigella spp.	24	3·9	1	0·3	14·35 (1·74, 118·25)	127	5·9	11	0·4	20·89 (10·68, 40·85)
Rotavirus	170	28	10	3·5	14·00 (6·80, 28·85)	557	26·0	155	5·1	10·53 (8·05, 13·78)
Adenovirus 40/41	20	3·3	3	1·0	3·66 (1·04, 12·94)	67	3·1	31	1·0	3·31 (2·08, 5·27)
St-ETEC	35	5·8	4	1·4	5·29 (1·81, 15·35)	99	4·6	57	1·9	2·76 (1·91, 3·99)
Cryptosporidium spp.	122	20	29	10·0	2·73 (1·68, 4·44)	348	16·0	217	7·1	2·76 (2·25, 3·39)
Cholera	10	1·6	1	0·3	7·01 (0·87, 56·15)	22	1·0	17	0·6	1·84 (0·93, 3·63)
Entamoeba spp.	17	2·8	4	1·4	2·25 (0·73, 6·94)	76	3·5	67	2·2	2·01 (1·39, 2·92)
Norovirus	39	6·4	27	9·3	0·76 (0·43, 1·34)	205	9·5	224	7·3	1·26 (1·02, 1·56)
Aeromonas	39	6·4	15	5·2	2·03 (0·99, 4·20)	131	6·1	129	4·2	1·80 (1·35, 2·40)
Campylobacter jejuni	58	9·5	35	12·0	0·67 (0·42, 1·06)	283	13·0	297	9·7	1·40 (1·17, 1·70)
EPEC-typical	71	12	24	8·3	1·72 (1·02, 2·91)	193	8·9	323	11	0·85 (0·79, 1·05)
12-23 months										
Shigella spp.	52	11	9	3·7	2·86 (1·36, 6·02)	432	16·0	96	2·3	9·62 (7·47, 12·39)
Cholera	15	3·1	4	1·7	1·75 (0·54, 5·34)	81	3·0	19	0·5	7·17 (4·16, 12·38)
Rotavirus	77	16	10	4·1	3·93 (1·95, 7·95)	495	18·0	163	3·9	7·11 (5·71, 8·85)
Adenovirus 40/41	10	2·1	5	2·1	0·96 (0·32, 2·94)	79	2·9	37	0·9	3·23 (2·15, 4·87)
St-ETEC	37	7·7	5	2·1	4·14 (1·57, 10·86)	133	4·9	97	2·3	2·25 (1·69, 2·99)
Cryptosporidium spp.	86	18	23	9·5	2·15 (1·28, 3·61)	288	11·0	253	6·1	1·92 (1·59, 2·33)
Aeromonas	34	7·1	12	5·0	2·17 (1·06, 4·44)	180	6·6	205	5·0	2·02 (1·59, 2·56)
Salmonella NT	12	2·5	8	3·3	0·70 (0·28, 1·78)	45	1·7	54	1·3	1·52 (1·01, 2·30)
Entamoeba spp.	15	3·1	9	3·7	0·83 (0·34, 2·02)	85	3·1	89	2·2	2·07 (1·48, 2·90)
24-59 months										
Cholera	9	8·5	0	0	--	92	4·4	23	0·6	12·14 (7·23, 20·38)
Shigella spp.	16	15	4	8·3	2·44 (0·71, 8·38)	409	19·0	90	2·4	14·21 (10·74, 18·80)
Rotavirus	11	10	0	0	--	148	7·1	91	2·4	3·69 (2·76, 4·95)
Salmonella NT	5	4·7	0	0	--	41	2·0	29	0·8	3·10 (1·8, 5·11)
Aeromonas	11	10	4	8·3	1·71 (0·49, 5·91)	179	8·5	206	5·4	2·84 (2·22, 3·63)
St-ETEC	7	6·6	0	0	--	81	3·9	64	1·7	2·21 (1·57, 3·14)
Entamoeba spp.	4	3·8	1	2·1	2·45 (0·25, 24·39)	60	2·9	68	1·8	1·81 (1·22, 2·67)
Campylobactor jejuni	15	14	7	15·0	1·06 (0·39, 2·90)	154	7·3	263	6·9	1·33 (1·06, 1·67)
Sapovirus	1	0·9	4	8·3	0·07 (0·01, 0·63)	54	2·6	101	2·6	0·98 (0·69, 1·40)

Abbreviations: rOR: Ratio of adjusted odds ratio, EPEC: Enteropathogenic Escherichia coli, ETEC: Enterotoxigenic Escherichia coli, Salmonella NT: Nontyphoidal Salmonella, Spp.: Species.

Supplementary Appendix 5:

Table 1: The effect of acute nutritional status on the associations between enteric pathogens and moderate-to-severe diarrhea on the relative (odd ratio) and absolute risk scales.

	**Estimated Interaction on Relative Risk[1] Logistic regression interaction terms**		**Estimated Interaction on Risk [1] RERI interaction terms**	
	**Interaction term (rOR)**	**95% CI**	**Interaction term (Absolute excess risk)**	**95% CI**
Viruses				
Adenovirus	0.8	0·3, 2·0	1·6	-2·7, 6·0
Norovirus	0·7	0·5, 1·1	-0·7	-1·7, 0·2
Rotavirus	1·0	0·6, 1·6	19·2	6·6, 31·8
Sapovirus	0·6	0·3, 1·0	-1·5	-2·9, -0·2
Bacteria				
Aeromonas	1·1	0·7, 2·0	1·0	-1·7, 3·7
Campylobacter jejuni	0·8	0·6, 1·2	-0·4	-1·8 1·0
Cholera	0·6	0·2, 1·9	5·8	-7·9, 19·5
EAEC	1·0	0·7, 1·3	0·1	-0·6, 0·8
EHEC	--	----	--	----
EPEC-atypical	0·7	0·4, 1·3	2·6	-0·0, 5·2
EPEC-typical	2·1	1·3, 3·3	12·0	1·4, 22·6
St-ETEC	2·6	1·2, 5·7	0·2	-1·1, 1·5
Lt-ETEC	1·2	0·7, 2·0	-1·1	-3·7, 1·5
Salmonella NT	0·6	0·3, 1·2	1·3	-9·4, 11·9
Shigella spp·	0·3	0·2, 0·5	2·6	-0·0, 5·2
Protozoa				
Cryptosporidium spp·	1·2	0·8, 1·8	3·5	0·8, 6·3
Entamoeba spp·	0·9	0·4, 1·8	0·5	-2·3, 3·3
Giardia	0·8	0·6, 1·1	-1·1	-1·7, -0·6

Abbreviations: EAEC: Enteroaggregative Escherichia coli, EHEC: Enterohemorrhagic Escherichia coli, EPEC: Enteropathogenic Escherichia coli, ETEC: Enterotoxigenic Escherichia coli, Salmonella NT: Nontyphoidal Salmonella, Spp.: Species.

[1]Adjusted age, pathogens associated with death during diarrhea (*Cryptosporidium*, *Entamoeba*, ST-ETEC & typical EPEC, and clustered by site.

Table 2: The effect of acute nutritional status on the associations between enteric pathogens and death during or after an episode of moderate-to-severe diarrhea on the relative (hazard ratio) and absolute risk scales.

	**Estimated Interaction on Relative Scale[1] Hazard ratio interaction terms**		**Estimated Interaction on Absolute Risk [1] RERI interaction terms**	
	**Interaction term (rHR)**	**95% CI**	**Interaction term (Absolute excess risk)**	**95% CI**
Viruses				
Adenovirus	0·92	0·15, 5·71	-1·48	-11·7, 28·76
Norovirus	0·24	0·08, 0·77	-8·30	-14·68, -1·91
Rotavirus	0·49	0·17, 1·39	-5·76	-10·27, -1·24
Sapovirus	0·57	0·11, 2·90	-4·36	-10·76, 2·04
Bacteria				
Aeromonas	--	----	-3·20	-8·60, 2·20
Campylobacter jejuni	0·73	0·18, 3·01	-7·56	-12·67, -2·44
Cholera	0·24	0·01, 5·10	-7·32	-18·76, 4·13
EAEC	1·13	0·58, 2·18	3·48	-2·60, 9·57
EHEC	--	-- --	--	----
EPEC-atypical	2·35	0·36, 15·30	4·08	-3·14, 11·31
EPEC-typical	1·27	0·64, 2·50	10·42	1·58, 19·26
St-ETEC	1·09	0·20, 5·96	3·62	-6·30, 13·54
Lt-ETEC	1·70	0·40, 7·27	-3·14	-6·05, -0·23
Salmonella NT	0·29	0·05, 1·66	-0·42	-13·35, 12·50
Shigella spp·	3·13	0·82, 11·91	5·35	-5·82, 16·52
Protozoa				
Cryptosporidium spp·	0·67	0·58, 0·78	3·62	-3·47, 10·72
Entamoeba spp·	0·97	0·35, 2·72	13·56	-3·89, 30·94
Giardia	1·50	0·45, 4·98	-4·07	-10·83, 2·70

Abbreviations: EAEC: Enteroaggregative Escherichia coli, EHEC: Enterohemorrhagic Escherichia coli, EPEC: Enteropathogenic Escherichia coli, ETEC: Enterotoxigenic Escherichia coli, Salmonella NT: Nontyphoidal Salmonella, Spp.: Species.
[1]Adjusted age, pathogens associated with death during diarrhea (*Cryptosporidium*, *Entamoeba*, ST-ETEC & typical EPEC, and clustered by site. [2]Crude Hazard ratios are also clustered by site.
